# Newborn screening reduces survival disparities in SCID after stem cell transplant: A PIDTC report

**DOI:** 10.70962/jhi.20250231

**Published:** 2026-07-07

**Authors:** Lena E. Winestone, Brent R. Logan, Xuerong Liu, Olatundun Williams, Elizabeth A. Dunn, Monica S. Thakar, Sharon A. Kidd, Talal Mousallem, Morna J. Dorsey, Richard J. O’Reilly, Neena Kapoor, Lisa Forbes Satter, Malika Kapadia, Soma C. Jyonouchi, Sharat Chandra, Christen L. Ebens, Deepakbabu Chellapandian, Sonali Chaudhury, Karin Chen, Blachy J. Dávila Saldaña, Ahmad Rayes, Troy C. Quigg, Shanmuganathan Chandrakasan, Jeffrey J. Bednarski, Kenneth B. DeSantes, Pierre Teira, Alfred P. Gillio, Hesham Eissa, Alan P. Knutsen, Victor M. Aquino, Evan B. Shereck, Theodore B. Moore, Emi H. Caywood, Mark T. Vander Lugt, Jacob Rozmus, Larisa Broglie, Lolie C. Yu, Ami J. Shah, Avni Y. Joshi, Rebecca H. Buckley, Jasmeen Dara, Joseph H. Oved, Hisham Abdel Azim, Caridad A. Martinez, Susan Prockop, Kathleen E. Sullivan, Jack J. Bleesing, Michael D. Keller, Lisa M. Madden, Suhag Parikh, Shalini Shenoy, Christine M. Seroogy, Tamar Rubin, Jennifer Licata, Jeffrey R. Andolina, Donald B. Kohn, Lauri Burroughs, Jennifer W. Leiding, Rebecca A. Marsh, Luigi D. Notarangelo, Sung-Yun Pai, Troy R. Torgerson, Michael A. Pulsipher, Jennifer Heimall, Geoffrey D.E. Cuvelier, Linda M. Griffith, Jennifer M. Puck, Christopher C. Dvorak, Morton J. Cowan, Elie Haddad

**Affiliations:** 1 Division of Pediatric Allergy, Immunology, and Blood and Marrow Transplantation, University of California, San Francisco, San Francisco, CA, USA; 2 University of California, San Francisco Benioff Children’s Hospitals, San Francisco, CA, USA; 3 Division of Biostatistics, Medical College of Wisconsin, Milwaukee, WI, USA; 4 Center for International Blood and Marrow Transplant Research, Milwaukee, WI, USA; 5 Clinical Research Division, Fred Hutchinson Cancer Center, Seattle, WA, USA; 6 Department of Pediatrics, University of Washington, Seattle, WA, USA; 7 Department of Pediatrics, Division of Pediatric Allergy and Immunology, Duke University School of Medicine, Durham, NC, USA; 8 Transplant & Cellular Therapy, MSK Kids, Memorial Sloan Kettering Cancer Center, New York, NY, USA; 9 Transplant and Cell Therapy Program and Laboratory, Department of Pediatrics, Keck School of Medicine, University of Southern California, Los Angeles, CA, USA; 10 Hematology, Oncology and Transplantation and Cellular Therapy, Children’s Hospital Los Angeles, Los Angeles, CA, USA; 11 Department of Pediatrics, Baylor College of Medicine, Houston, TX, USA; 12 Immunology Allergy and Retrovirology, Center for Human Immunobiology, Texas Children’s Hospital Infusion Center, Houston, TX, USA; 13 Department of Pediatrics, Harvard University Medical School, Boston, MA, USA; 14 Dana Farber/Boston Children’s Cancer and Blood Disorders Center, Boston, MA, USA; 15 Division of Allergy and Immunology, Children’s Hospital of Philadelphia, Philadelphia, PA, USA; 16 Department of Pediatrics, University of Cincinnati College of Medicine, Cincinnati, OH, USA; 17 Division of Bone Marrow Transplantation and Immune Deficiency, Cincinnati Children’s Hospital Medical Center, Cincinnati, OH, USA; 18 Department of Pediatrics, Division of Blood and Marrow Transplantation & Cellular Therapy, University of Minnesota, Minneapolis, MN, USA; 19 Cancer and Blood Disorders Institute, Johns Hopkins All Children’s Hospital, St. Petersburg, FL, USA; 20 Northwestern University Feinberg School of Medicine, Chicago, IL, USA; 21 Division of Pediatric Hematology/Oncology/Stem Cell Transplantation, Ann and Robert H. Lurie Children’s Hospital of Chicago, Chicago, IL, USA; 22 Seattle Children’s Research Institute, Seattle, WA, USA; 23 Department of Pediatrics, George Washington University School of Medicine and Health Sciences, Washington, DC, USA; 24 Center for Cancer and Immunology Research, Children’s National Hospital, Washington, DC, USA; 25 Pediatric Immunology and Hematopoietic Cell Transplantation/Cellular Therapy Program, Primary Children’s Hospital, University of Utah, Salt Lake City, UT, USA; 26 Department of Pediatrics, Michigan State University College of Human Medicine, Grand Rapids, MI, USA; 27 Pediatric Bone Marrow Transplantation and Cellular Therapy Program, Helen DeVos Children’s Hospital, Grand Rapids, MI, USA; 28 Department of Pediatrics, Emory University School of Medicine, Atlanta, GA, USA; 29 Aflac Cancer and Blood Disorders Center, Children’s Healthcare of Atlanta, Atlanta, GA, USA; 30 Department of Pediatrics, Washington University in St. Louis School of Medicine, St. Louis, MO, USA; 31 Stem Cell & Regenerative Medicine Center, University of Madison-Wisconsin, Madison, WI, USA; 32 Department of Pediatrics, Department of Microbiology, Immunology and Infectious Diseases, Centre de Recherche Azrieli du CHU Sainte-Justine, University of Montreal, Montreal, Canada; 33 Hematology-Oncology Department, Centre Hospitalier Universitaire Sainte-Justine, Montreal, Canada; 34 Pediatric Stem Cell and Cellular Therapy Division, Joseph M. Sanzari Children’s Hospital at Hackensack Meridian Health Hackensack University Medical Center, Hackensack, NJ, USA; 35 Department of Pediatrics, University of Colorado, Aurora, CO, USA; 36 Bone Marrow Transplant and Cellular Therapeutics, Children’s Hospital of Colorado, Aurora, CO, USA; 37 Pediatric Allergy and Immunology, St. Louis University, St. Louis, MO, USA; 38 Jeffrey Modell Diagnostic & Research Center for Primary Immunodeficiencies, Cardinal Glennon Children’s Hospital, St. Louis, MO, USA; 39 Department of Pediatrics, University of Texas Southwestern Medical Center Dallas, Dallas, TX, USA; 40 Department of Pediatrics, Oregon Health & Science University, Portland, OR, USA; 41 Pediatric Blood and Marrow Transplant Program, Division of Pediatric Hematology/Oncology in the Department of Pediatrics, University of California, Los Angeles, Los Angeles, CA, USA; 42 Sidney Kimmel Medical College, Thomas Jefferson University, Philadelphia, PA, USA; 43 Transplant and Cellular Therapy, Lisa Dean Moseley Foundation Institute of Cancer and Blood Disorders, Nemours Children’s Health, Delaware, Wilmington, DE, USA; 44 Department of Pediatrics, University of Michigan, Ann Arbor, MI, USA; 45 Department of Pediatrics, University of British Columbia, Vancouver, Canada; 46 BC Children’s Hospital, Vancouver, Canada; 47 Department of Pediatrics, Medical College of Wisconsin, Milwaukee, WI, USA; 48 Louisiana State University Health New Orleans School of Medicine, New Orleans, LA, USA; 49 Children’s Hospital of New Orleans, New Orleans, LA, USA; 50 Division of Hematology/Oncology/Stem Cell Transplantation and Regenerative Medicine, Department of Pediatrics, Stanford School of Medicine, Palo Alto, CA, USA; 51 Division of Pediatric Allergy/Immunology, Mayo Clinic Children’s Center, Rochester, MN, USA; 52 Loma Linda University School of Medicine, Cancer Center, Children Hospital and Medical Center, Loma Linda, CA, USA; 53 Cell Therapy and Bone Marrow Transplant Division, Department of Pediatrics, Baylor College of Medicine, Houston, TX, USA; 54 Department of Pediatrics, Perelman School of Medicine at the University of Pennsylvania, Philadelphia, PA, USA; 55 Pediatric Bone Marrow Transplant Program, University of California, Davis, Sacramento, CA, USA; 56 Department of Pediatrics, University of Wisconsin School of Medicine and Public Health, Madison, WI, USA; 57 Division of Pediatric Clinical Immunology and Allergy, Department of Pediatrics and Child Health, University of Manitoba, Winnipeg, Canada; 58 Winnipeg Children’s Hospital, Winnipeg, Canada; 59 Department of Pediatrics, Golisano Children’s Hospital, University of Rochester Medical Center, Rochester, NY, USA; 60 Division of Allergy and Immunology, Department of Pediatrics, Johns Hopkins University, Baltimore, MD, USA; 61 Institute for Clinical and Translational Research, Johns Hopkins All Children’s Hospital, St. Petersburg, FL, USA; 62 Laboratory of Clinical Immunology and Microbiology, National Institute of Allergy and Infectious Diseases/National Institutes of Health, Bethesda, MD, USA; 63 Immune Deficiency Cellular Therapy Program, Center for Cancer Research, National Cancer Institute/National Institutes of Health, Bethesda, MD, USA; 64 Allen Institute for Immunology, Seattle, WA, USA; 65 Division of Pediatric Hematology and Oncology, Intermountain Primary Children’s Hospital, Salt Lake City, UT, USA; 66 Huntsman Cancer Institute, Spencer Fox Eccles School of Medicine at the University of Utah, Salt Lake City, UT, USA; 67 Pediatric Oncology and Transplantation, Alberta Children’s Hospital, University of Calgary, Calgary, Canada; 68 Division of Allergy, Immunology and Transplantation, National Institute of Allergy and Infectious Diseases/National Institutes of Health, Bethesda, MD, USA

## Abstract

Black race and Hispanic ethnicity are associated with higher mortality in severe combined immunodeficiency (SCID) following hematopoietic cell transplantation (HCT), though mechanisms remain unclear. We evaluated 796 children with SCID who received nonsibling HCT between 1982 and 2020 using data from the Primary Immune Deficiency Treatment Consortium. Overall survival for Black (aHR 2.47, 95%CI 1.64, 3.71) and Asian/Pacific Islander patients (aHR 1.82, 95%CI 1.00, 3.30) was significantly lower compared with non-Hispanic White patients, while Hispanic patients had lower event-free survival (aHR 1.83, 95%CI 1.27, 2.63) compared with non-Hispanic White patients. Even after adjusting for age and infection, Black patients with SCID had more than twofold hazard of death compared with non-Hispanic White patients. NBS was associated with earlier diagnosis, reduced infection at HCT, and elimination of survival disparities between Black and non-Hispanic White patients. These findings suggest that universal, system-level interventions such as NBS can mitigate disparities in outcomes for children with SCID.

## Teaser

Winestone et al. demonstrate that in 796 children with SCID transplanted with nonsibling donors between 1982 and 2020, survival was 18.8% lower among Black patients, 14.4% lower among Asian/Pacific Islander patients, and 6.9% lower among Hispanic patients compared with non-Hispanic White patients (P < 0.01). Newborn screening for SCID mitigates wide disparities between Black and non-Hispanic White patients.

## Introduction

Severe combined immunodeficiency (SCID) comprises a heterogeneous group of genetic disorders of lymphopoiesis (together with metabolic toxicity in the context of adenosine deaminase [ADA] deficiency) characterized by lack of adaptive immune responses and is fatal if untreated. The Primary Immune Deficiency Treatment Consortium (PIDTC) conducted a natural history study of patients treated for SCID in both pre- and postintroduction of newborn screening (NBS) (1). Prior to population-based NBS for SCID, SCID was typically diagnosed after the development of infection or by prenatal/newborn testing for the small group of families with a previously affected child. Allogeneic hematopoietic cell transplantation (HCT) is the standard of care for SCID. Although human leukocyte antigen (HLA)–matched sibling donors (MSD) are ideal donors, alternative donor transplants can lead to favorable outcomes with appropriate conditioning. As demonstrated by PIDTC (2, 3) and other studies (4), key features consistently associated with favorable outcomes are the absence of active infections and younger age at HCT.

NBS for SCID was piloted in 2008 and recommended by U.S. Secretary of Health and Human Services for inclusion in state NBS panels in 2010 (5, 6). By 2018, all 50 U.S. states adopted SCID NBS (7, 8). With increasing adoption, survival following HCT for SCID notably improved in North America (9).

Race is a known predictor of HCT outcome, due to difficulties in finding HLA-matched donors and factors independent of HCT donor, stem cell source, and match quality, including health insurance type, distance to care, and socioeconomic status (SES) (10, 11). It was unclear whether these data were applicable to patients with SCID, given its rarity among HCT indications. Using PIDTC data, we showed that Black race and Hispanic ethnicity were associated with higher mortality among patients with SCID who received HCT from nonsibling donors compared with non-Hispanic (NH) White patients (3).

Building on that finding, we investigated the etiology of racial and ethnic disparities in survival of post-HCT patients with SCID. We hypothesized that Black patients undergoing HCT for SCID had a higher incidence of infection pretransplant and greater age at transplant due to delays in diagnosis. We further hypothesized that progressive implementation of NBS in North America reduced racial and ethnic disparities in survival after HCT by shortening the time to transplant and reducing pre-HCT infection.

## Results

### Survival disparities by race and ethnicity

**Figure S1.**
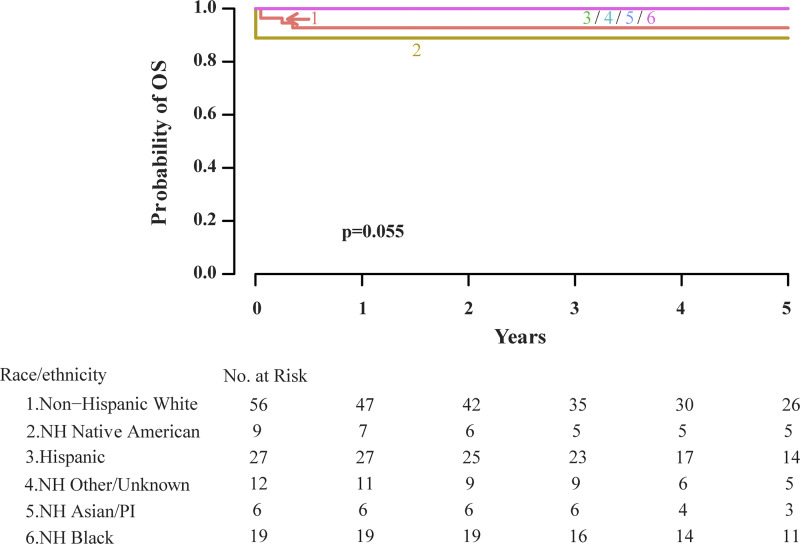
**OS among MSD transplants.** (1) (red) NH White, (2) (yellow) NH Native American, (3) (green) Hispanic, (4) (teal) unknown or other race/ethnicity, (5) (blue) NH Asian/PI, and (6) (pink) NH Black. No statistical differences in OS are detected among MSD transplants for SCID (P = 0.055).

**Figure S2.**
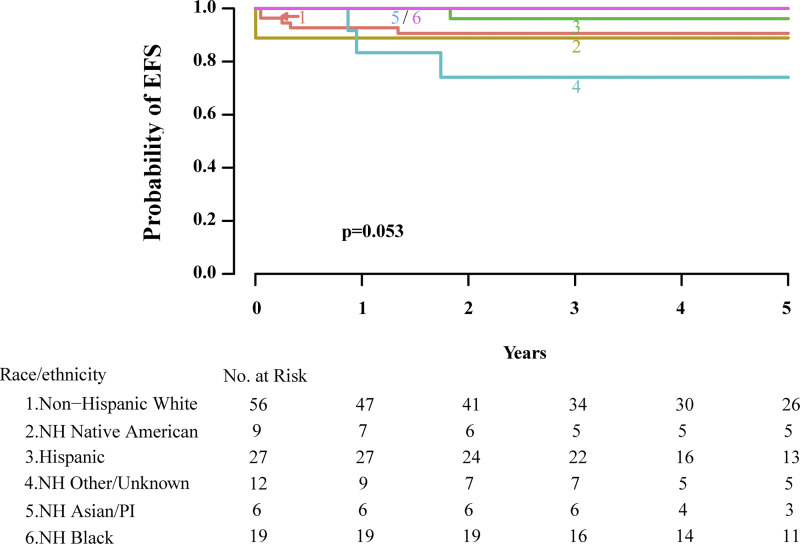
**EFS among MSD transplants.** (1) (red) NH White, (2) (yellow) NH Native American, (3) (green) Hispanic, (4) (teal) unknown or other race/ethnicity, (5) (blue) NH Asian/PI, and (6) (pink) NH Black. No statistical differences in EFS are detected among MSD transplants for SCID (P = 0.053).

Overall survival (OS) among the 14% of patients who received MSD HCT ranged from 88 to 100% with no statistically significant differences by race and ethnicity (P = 0.06, Fig. S1). Among recipients of MSD HCT, there was a suggestion of worse event-free survival (EFS) among the small number of NH Other/Unknown patients (*n* = 12) compared with the NH White patients (P = 0.05, Fig. S2). Given uniformly favorable outcomes across racial groups receiving MSD HCT, these were excluded from subsequent analyses.

**Figure 1.**
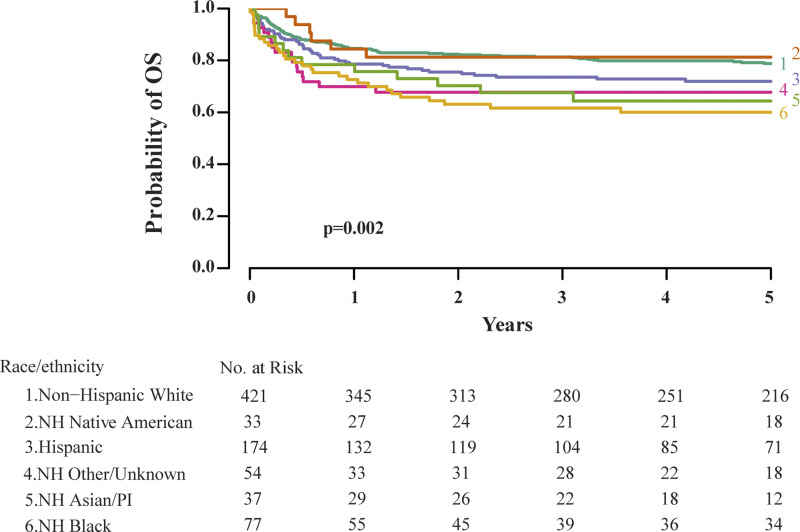
**Overall survival for patients who received non-sibling stem cell transplants for SCID by race and ethnicity.** (1) (green) NH White, (2) (red) NH Native American, (3) (indigo) Hispanic, (4) (pink) unknown or other race/ethnicity, (5) (light green) NH Asian/PI, and (6) (yellow) NH Black. NH White and Native American patients demonstrate the highest OS, and Black, Other, and Asian/PI patients demonstrate the lowest OS (P = 0.002).

Multivariable models to compare survival by race and ethnicity included age at HCT, conditioning, donor type, SCID genotype, infection status, and decade of HCT (Table 1). OS among Black (adjusted hazard ratio [aHR] 2.47, 95% confidence interval [95%CI] 1.64, 3.71, P < 0.001) and Asian/Pacific Islander (PI) patients (aHR 1.82, 95%CI 1.00, 3.30) who received transplants from nonsiblings was lower compared with NH White patients. OS among Hispanic patients also seemed lower compared with NH White patients, although this difference was not statistically significant (aHR 1.32, 95%CI 0.93, 1.88; Fig. 1 and Table 2). OS for Native American and NH White patients was similar (aHR 0.74, 95%CI 0.35, 1.56). EFS also differed by race and ethnicity (P = 0.006) with lower EFS among Hispanic patients (aHR 1.83, 95%CI 1.27, 2.63; Table S1). Causes of death by race and ethnicity (Table S2) showed infection as the most common cause of death (36–64% of deaths) with no statistically significant differences by race and ethnicity (P = 0.54).

**Table 1.** Multivariable model of OS for non-MSD transplants

Category	Variable	HR (95%CI)	P value	Overall P value
Race/Ethnicity	NH White	ref	​	<0.001
	Hispanic	1.32 (0.93, 1.88)	0.12	​
	Asian/PI	1.82 (1.00, 3.30)	**0.05**	​
	Black	2.47 (1.64, 3.71)	**<0.001**	​
	Native American	0.74 (0.35, 1.56)	0.43	​
	Unknown	1.95 (1.19, 3.08)	**0.008**	​
Age at treatment	<3.5 mo	ref	​	0.002
	>3.5 mo	1.92 (1.27, 2.91)	**0.002**	​
Donor Type^a^	HLA-mismatched relative	ref	​	0.26
	HLA-matched other relative	0.47 (0.20, 1.07)	0.08	​
	HLA-matched unrelated	1.17 (0.69, 1.98)	0.55	​
	HLA-mismatched unrelated	1.26 (0.85, 1.88)	0.25	​
	Unrelated/unknown matching	0.80 (0.25, 2.58)	0.71	​
Infection status	No infection	ref	​	<0.001
	Active infection	2.11 (1.35, 3.29)	**0.001**	​
	Missing	0.34 (0.05, 2.56)	0.30	​
	Resolved infection	1.14 (0.69, 1.88)	0.61	​
	Unknown infection status	1.78 (1.02, 3.11)	**0.03**	​
Genotype	*IL2RG/JAK3*	ref	​	<0.001
	*ADA*	2.33 (1.29, 4.18)	**0.005**	​
	*DCLRE1C*	4.04 (2.09, 7.81)	**<0.001**	​
	*IL7R*	1.27 (0.69, 2.33)	0.540	​
	Novel/missing	2.34 (1.65, 3.31)	**<0.001**	​
	Other/unknown	3.35 (1.65, 7.23)	**0.002**	​
	*RAG1/2*	2.18 (1.28, 3.73)	**0.004**	​
Decade of treatment	1980–1990	ref	​	0.02
	1990–2000	1.33 (0.89, 2.04)	0.18	​
	2000–2010	1.16 (0.76, 1.85)	0.51	​
	2010–2020	0.61 (0.33, 1.08)	0.11	​

HLA, human leukocyte antigen; Ref, reference group. Bold values indicate P values that met the threshold for statistical significance.

a In initial model building where MSD transplants were included, donor type was statistically significant; thus, donor type was retained in the final model (despite the subsequent exclusion of patients who received a transplant from an MSD) based on the previous statistical significance and the effect detected of HLA-matched other relative donor type.

**Table 2.** Overall survival in patients with SCID who received non-MSD HCT

Outcome	NH White, *N* = 421	Hispanic, *N* = 174	Black, *N* = 77	Asian/PI, *N* = 37	Native American, *N* = 33	Unknown/Other, *N* = 54	P value
OS							<0.01
Unadjusted HR for OS (95%CI)	ref	1.34 (0.94, 1.90)	2.43 (1.62, 3.65)	1.79 (0.98, 3.26)	0.74 (0.35, 1.59)	2.00 (1.22, 3.28)	​
1-year OS	84.6 (80.8–87.8)	78.6 (71.7–84.0)	72.6 (61.2–81.2)	78.4 (61.4–88.6)	84.4 (66.5–93.2)	69.9 (55.6–80.3)	​
5-year OS	78.8 (74.4–82.5)	71.9 (64.4–78.2)	60.0 (47.9–70.1)	64.4 (46.5–77.6)	81.3 (63.0–91.1)	67.8 (53.3–78.6)	​

### Survival disparities stratified by trigger for diagnosis

**Figure 2.**
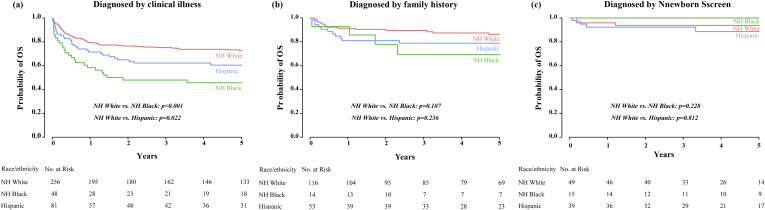
**Overall survival by race and ethnicity stratified by diagnosis trigger. (a)** Diagnosed by clinical illness. **(b)** Diagnosed by family history. **(c)** Diagnosed by newborn screen. Red represents NH White patients, blue represents Hispanic patients, and green represents NH Black patients. While there is a significant difference among those diagnosed by clinical illness (a, P = 0.022, P < 0.001, respectively), there is no statistical difference among those diagnosed by FH (b) or NBS (c).

To test whether NBS reduced observed disparities through earlier detection, we stratified by trigger for diagnosis. Across the entire cohort, 56% of diagnoses were triggered by clinical illness, 28% were diagnosed based on family history (FH), and 16% were diagnosed by NBS. The introduction of NBS resulted in improvement in 5-year OS for all patients who received nonsibling transplants; the greatest benefit was observed in Black patients diagnosed by NBS (Fig. 2; 5-year OS: NBS 100% vs. clinical illness 46%, P < 0.01).

**Figure S3.**
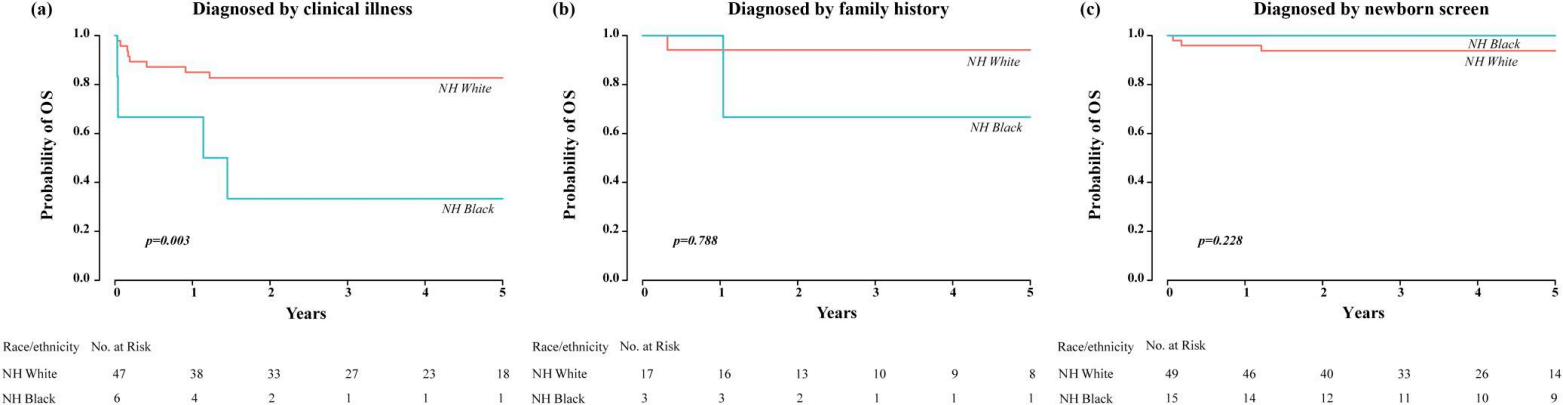
**Sensitivity analyses evaluating overall survival stratified by diagnosis trigger restricted to the contemporary era.** **(a)** Diagnosed by clinical illness. **(b)** Diagnosed by family history. **(c)** Diagnosed by newborn screen. Red represents NH White patients, and teal represents NH Black patients. While there is a significant difference among those diagnosed by clinical illness (a, P = 0.003), there is no statistical difference among those diagnosed by FH (b) or NBS (c).

Survival analyses stratified by diagnosis trigger demonstrated differences in OS between Black, Hispanic, and NH White patients diagnosed by clinical illness (46% vs. 67% vs. 73%, P < 0.001 and P = 0.02; Fig. 2 a). On the other hand, differences by FH (69% vs. 87%, P = 0.11; Fig. 2 b) or NBS (100% vs. 94%, P = 0.23; Fig. 2 c) were not statistically significant. Sensitivity analysis restricted to patients diagnosed between 2010 and 2020 revealed disparities persisted between Black and White patients diagnosed by clinical illness (33% vs. 83%, P = 0.003; Fig. S3, a–c). However, these findings should be interpreted with caution given the very low number of Black patients in this restricted timeframe. OS was similar in Hispanic and NH White patients diagnosed by FH (81% vs. 87%; P = 0.24; Fig. 2 b) or NBS (89% vs. 94%; P = 0.81; Fig. 2 c). While there were relatively few Asian/PI patients, the survival disparity persisted even with NBS (5-year OS: 50% vs. 94%, P = 0.001; data not shown).

Patients diagnosed due to clinical illness uniformly underwent treatment later (median 226 days vs. 95 days, P < 0.001) and more frequently had an active infection at HCT (51% vs. 21%, P < 0.001) than those diagnosed by NBS. Disparities in median age at HCT were noted among those who received a non-MSD transplant diagnosed by FH (White: 54.5 days, Hispanic: 95 days, Black: 105.5 days; P = 0.04, Table 3), whereas there were no age differences by race or ethnicity among those diagnosed by clinical illness or NBS. Differences in prevalence of active infection at the time of HCT were also notable when diagnosis was triggered by FH where 16% of NH White patients, 29% of Black patients, and 50% of Native American patients came to HCT with active infection (P = 0.001).

**Table 3.** Time to treatment and pre-existing infection by race and ethnicity among non-MSD transplants stratified by method of diagnosis (clinical illness vs. NBS vs. FH)

Diagnosed by clinical illness	Total, *N* = 444	NH White, *N* = 256	Hispanic, *N* = 81	Black, *N* = 48	Asian/PI, *N* = 18	Native American, *N* = 17	Unknown, *N* = 24	P value
**Age at treatment**	​							0.24
Median (days) (min–max)	226 (13–5,137)	225 (13–5,137)	247 (83–3,952)	225.5 (59–1309)	209.5 (101–3,631)	181 (57–634)	271.5 (39–1,197)	​
**Baseline infection**	​							0.12
Active infection	221 (50.6%)	119 (47.6%)	44 (55.0%)	27 (56.3%)	12 (66.7%)	8 (47.1%)	11 (45.8%)	​
No infection	37 (8.5%)	27 (10.8%)	5 (6.3%)	3 (6.3%)	0 (0.0%)	2 (11.8%)	0 (0.0%)	
Resolved infection	122 (27.9%)	73 (29.2%)	24 (30.0%)	14 (29.2%)	3 (16.7%)	3 (17.6%)	5 (20.8%)	
Unknown/missing	64 (14.4%)	37 (14.5%)	8 (9.9%)	4 (8.3%)	3 (16.7%)	4 (23.5%)	8 (33.3%)	​

Diagnosed by NBS								
Variables	Total, *N* = 129	NH White, *N* = 49	Hispanic, *N* = 39	Black, *N* = 15	Asian/PI, *N* = 8	Native American, *N* = 6	Unknown, *N* = 12	P value
**Age at treatment**	​							0.99
Median (days) (min–max)	95 (26–251)	94 (26–222)	99 (45–238)	88 (60–251)	105.5 (56–124)	96 (61–133)	94 (50–160)	​
**Baseline infection**	​							0.12
Active infection	27 (20.9%)	3 (6.1%)	11 (28.2%)	2 (13.3%)	2 (25.0%)	4 (66.7%)	5 (41.7%)	​
No infection	87 (67.4%)	38 (77.6%)	24 (61.5%)	11 (73.3%)	5 (62.5%)	2 (33.3%)	7 (58.3%)	
Resolved infection	14 (10.9%)	7 (14.3%)	4 (10.3%)	2 (13.3%)	1 (12.5%)	0 (0.0%)	0 (0.0%)	
Unknown/missing	1 (0.8%)	1 (2.0%)	0 (0.0%)	0 (0.0%)	0 (0.0%)	0 (0.0%)	0 (0.0%)	​

Diagnosed by family history								
Variables	Total, *N* = 222	NH White, *N* = 116	Hispanic, *N* = 53	Black, *N* = 14	Asian/PI, *N* = 11	Native American, *N* = 10	Unknown, *N* = 18	P value
**Age at treatment**	​							0.04
Median (days) (min–max)	72 (7–2,050)	54.5 (7–1,314)	95 (16–2,050)	105.5 (20–443)	67 (24–631)	74.5 (33–314)	124 (28–401)	​
**Baseline infection**								0.001
Active infection	41 (18.9%)	18 (15.8%)	10 (19.2%)	4 (28.6%)	1 (9.1%)	5 (50.0%)	3 (18.8%)	​
No infection	114 (52.5%)	63 (55.3%)	26 (50.0%)	8 (57.1%)	10 (90.9%)	2 (20.0%)	5 (31.3%)	
Resolved infection	43 (19.8%)	23 (20.2%)	14 (26.9%)	2 (14.3%)	0 (0.0%)	2 (20.0%)	2 (12.5%)	
Unknown/Missing	24 (10.8%)	12 (10.3%)	3 (5.7%)	0 (0.0%)	0 (0.0%)	1 (10.0%)	8 (44.4%)	​

### Variation in patient and transplant characteristics by race and ethnicity

Of 925 patients who received HCT (including from MSD), 52% were NH White, 22% were Hispanic, 10% were Black, 4.6% were Asian/PI, and 4.5% were Native American (Table 4). 39% of both Black and Hispanic patients had an active infection at HCT compared with 34% of NH White patients (P = 0.52). Maternal engraftment and autoimmune cytopenias were most common in the Asian/PI population (44% and 12%, respectively, Table 4).

**Table 4.** Patient characteristics of entire cohort (including MSD transplant recipients)

Characteristics	Total, *N* = 925	NH White, *N* = 477	Hispanic, *N* = 201	Black, *N* = 96	Asian/PI, *N* = 43	Native American, *N* = 42	Other/Unknown, *N* = 66	P value
Male sex	641 (69.3%)	345 (72.3%)	130 (64.7%)	73 (76.0%)	32 (74.4%)	14 (33.3%)	47 (71.2%)	**<0.001**
Median age at diagnosis, days (min–max)	118 (−17–4,918)	136 (0–4,918)	100 (0–4,636)	137 (0–907)	48 (0–3,084)	71 (1.0–526)	106.5 (−17–2,269)	0.09
Median age at treatment, days (min–max)	167 (0–7,067)	176 (7–5,137)	146 (13–4,783)	188 (12–1,309)	124 (17–3,631)	128 (0–634)	153 (16–7,067)	0.55
**Age at treatment**								0.42
<3.5 mo	303 (32.8%)	149 (31.2%)	64 (31.8%)	30 (31.3%)	17 (39.5%)	19 (45.2%)	24 (36.4%)	​
>3.5 mo	622 (67.2%)	328 (68.8%)	137 (68.2%)	66 (68.8%)	26 (60.5%)	23 (54.8%)	42 (63.6%)	
**Decade of treatment**								**<0.001**
1982–1990	115 (12.4%)	70 (14.7%)	11 (5.5%)	14 (14.6%)	3 (7.0%)	6 (14.3%)	11 (16.7%)	​
1990–2000	224 (24.2%)	132 (27.7%)	37 (18.4%)	19 (19.8%)	6 (14.0%)	16 (38.1%)	14 (21.2%)	
2000–2010	296 (32.0%)	142 (29.8%)	82 (40.8%)	32 (33.3%)	14 (32.6%)	10 (23.8%)	16 (24.2%)	
2010–2020	290 (31.4%)	133 (27.9%)	71 (35.3%)	31 (32.3%)	20 (46.5%)	10 (23.8%)	25 (37.9%)	
**Trigger**								**0.03**
Family history	264 (28.6%)	134 (28.1%)	59 (29.5%)	21 (21.9%)	14 (32.6%)	14 (33.3%)	22 (33.3%)	​
Clinical illness	515 (55.7%)	288 (60.4%)	98 (49.0%)	56 (58.3%)	20 (46.5%)	22 (52.4%)	31 (47.0%)	
NBS	145 (15.7%)	55 (11.5%)	43 (21.5%)	19 (19.8%)	9 (20.9%)	6 (14.3%)	13 (19.7%)	
Missing	1 (0.1%)	0 (0.0%)	1 (0.5%)	0 (0.0%)	0 (0.0%)	0 (0.0%)	0 (0.0%)	​
**Baseline infection**								**<0.001**
Active infection	336 (36.9%)	160 (34.3%)	78 (39.2%)	37 (38.5%)	17 (40.5%)	20 (47.6%)	24 (37.5%)	​
No infection	271 (29.8%)	143 (30.6%)	60 (30.2%)	27 (28.1%)	18 (42.9%)	8 (19.0%)	15 (23.4%)	
Resolved infection	209 (23.0%)	115 (24.6%)	50 (25.1%)	26 (27.1%)	4 (9.5%)	6 (14.3%)	8 (12.5%)	
Unknown infection status	94 (10.3%)	49 (10.5%)	11 (5.5%)	6 (6.3%)	3 (7.1%)	8 (19.0%)	17 (26.6%)	
Missing	15 (1.6%)	10 (2.1%)	2 (1.0%)	0 (0.0%)	1 (2.3%)	0 (0.0%)	2 (3.0%)	​
**Maternal engraftment**	164 (26.5%)	71 (21.0%)	48 (35.0%)	15 (23.4%)	11 (44.0%)	9 (40.9%)	10 (30.3%)	**0.004**
**Maternal GVHD**	49 (5.4%)	19 (4.1%)	18 (9.0%)	3 (3.2%)	2 (4.8%)	5 (12.2%)	2 (3.1%)	**0.03**
**Autoimmune cytopenia**	72 (7.9%)	33 (7.0%)	17 (8.5%)	8 (8.3%)	5 (11.9%)	3 (7.3%)	6 (9.4%)	0.20
**Genotype**								**<0.001**
*ADA*	55 (5.9%)	33 (6.9%)	9 (4.5%)	7 (7.3%)	0 (0.0%)	2 (4.8%)	4 (6.1%)	​
*DCLRE1C*	39 (4.2%)	11 (2.3%)	5 (2.5%)	0 (0.0%)	3 (7.0%)	16 (38%)	4 (6.1%)	
*IL2RG*	274 (30%)	157 (33%)	41 (20%)	41 (43%)	21 (49%)	4 (9.5%)	10 (15%)	
*IL7R*	67 (7.2%)	26 (5.5%)	31 (15%)	3 (3.1%)	1 (2.3%)	1 (2.4%)	5 (7.6%)	
*JAK3*	40 (4.3%)	19 (4.0%)	10 (7.2%)	8 (8.3%)	2 (4.7%)	0 (0.0%)	1 (1.5%)	
*RAG1*	80 (8.6%)	41 (8.6%)	17 (8.5%)	6 (6.3%)	3 (7.0%)	6 (14%)	7 (11%)	
*RAG2*	35 (3.8%)	10 (2.1%)	16 (8.0%)	0 (0.0%)	2 (4.7%)	0 (0.0%)	7 (11%)	
Other/unknown	335 (36%)	180 (38%)	72 (36%)	31 (32%)	11 (26%)	13 (31%)	28 (42%)	​

GVHD, graft-versus-host disease. Bold values indicate P values that met the threshold for statistical significance.

SCID genotype varied significantly by race and ethnicity (P < 0.001). *IL2RG* was the most common genotype and was particularly common in both Black (43%) and Asian/PI (49%) populations, whereas most Native American patients had *DCLRE1C* (38%) or *RAG1* (14%) genotypes. *IL7R* was more prevalent in the Hispanic population (15%) than in the overall cohort (7%).

HLA-mismatched related donors were most used across all groups (Table S3) with notably high utilization among Black and Native American patients at 55% and 62%, respectively. Hispanic patients had the highest use of myeloablative conditioning at 33%, while Native Americans were most likely to receive immunosuppression only as a preparative approach and Black patients were the most likely to receive unconditioned transplants. Serotherapy was least utilized for Native American patients (38%).

### Differences in subsequent HCT, graft-vs.-host disease (GVHD), and immune reconstitution by race and ethnicity

Subsequent HCT was required in 16% of patients with no differences by race or ethnicity. The cumulative incidence of acute grade III–IV GVHD at Day 180 was 10%, and the cumulative incidence of chronic GVHD at 2 years was 15% (Table S2). There was no difference in GVHD by race or ethnicity, though there was a notably low rate of chronic GVHD in the Native American population (3.5%). Native American patients also had poorer T and B cell immune reconstitution after transplant compared with patients in other groups, likely due to the *DCLRE1C* genotype and avoidance of alkylators in conditioning. Median B cell count 6 mo after HCT among Native American patients was 2 cells/μl compared with 410 cells/μl for NH White patients (P < 0.001), and median naïve T cell count was 0 for Native American patients compared with 216 cells/μl for NH White patients (P = 0.02, Table S4 a and b).

## Discussion

In this large North American cohort of 925 patients with SCID, we demonstrated stark racial and ethnic disparities in survival among the 796 patients who received a nonsibling HCT, with Black and Asian/PI patients having substantially worse OS compared with NH White patients. Interestingly, age and infection status at HCT did not differ significantly by race and ethnicity overall. While not in the setting of a randomized clinical trial and with relatively small racial subgroups, we observed that NBS for SCID eliminated the survival disparity between Black and NH White patients. This effect was not attributable to other changes in HCT care over time given that the survival disparity persisted into the contemporary era among the small number of patients who were still diagnosed by clinical illness (rather than NBS). These observations strongly suggest that systematic and universal policy-level practice changes can ameliorate the SCID survival disparities associated with race and ethnicity, although they likely do not fully eliminate the role of health insurance, distance to care, and SES. NBS allows for the elimination of delays in time to transplant, and our data suggest that racial and ethnic disparities are most notable within the population of those with late presentation and/or diagnosis.

Long-standing racial and ethnic disparities in infant mortality for many diseases persist into the contemporary era (12). Similarly, racial and ethnic disparities in mortality following HCT for malignant conditions such as leukemia, myelodysplastic syndrome, and multiple myeloma are well described in adults (10, 13). We noted race and ethnicity as risk factors for higher posttransplant mortality in SCID in a prior PIDTC publication (3). Using an expanded PIDTC cohort, we explored the potential drivers of this disparity, accounting for differences in donor type and conditioning. While we hypothesized that access to care, as measured by time from birth to diagnosis and birth to HCT, would be an important contributor to the survival disparity, differences in HCT timing by race and ethnicity were noted only among those diagnosed by FH, with the median time to HCT for Black patients almost double that of NH White patients. We also hypothesized that infection prior to HCT, another known predictor of survival in patients with SCID, would vary by race and ethnicity. But notably, meaningful differences in active infection were largely limited to the 28% of patients diagnosed by FH. This substantial racial and ethnicity variability among those diagnosed by FH was surprising and may be attributable to the responsibility being placed primarily on the family and primary care rather than a systematic and organized program designed for early detection such as NBS. Furthermore, while we did not find quantitative differences in age at HCT or proportion with active infection coming into HCT, there may be qualitative differences in infection burden/type and the care received. We hypothesize that potential unmeasured contributors including other SES metrics, center-level variation, insurance-related delays, donor availability, and differential access to early specialized evaluation likely drive the disparities detected.

The differences in OS seen between Black and White patients are particularly striking since more Black patients had SCID caused by variants in *IL2RG* or *JAK3* (51%) compared with NH White patients (37%); *IL2RG/JAK3* genotypes generally have the best posttransplant outcomes of all SCID genotypes (3, 9). Considering these genotypic differences, the outcomes of the Black population were relatively worse than represented by their 60% 5-year OS. More Asian/PI patients had variants in *IL2RG* compared with NH White patients and yet also had lower OS (64% vs. 79%, P = 0.05). Asian/PI patients had the highest prevalence of maternal engraftment and autoimmune cytopenias, conditions that may complicate posttransplant recovery. Asian/PI patients were the most likely to receive umbilical cord grafts and were the most likely to receive conditioning. This finding aligns with data demonstrating reduced adult unrelated donor availability for Asian patients and improved access through cord blood transplantation, which requires less stringent HLA matching and offers greater ethnic diversity compared with adult donor registries (14). Hispanic patients, despite having a higher prevalence of the relatively favorable *IL7R* genotype, exhibited lower EFS (P = 0.001) compared with NH White patients.

The favorable OS among Native American patients with SCID who received non-MSD transplants is surprising given the predominance of *DCLRE1C* in the Navajo Nation, the largest tribal nation in the United States. *DCLRE1C* is generally associated with the worst prognosis among SCID genotypes (3). There are several potential explanations for this unexpectedly favorable outcome. NBS was piloted early on the Navajo reservation (15) due to a known founder mutation and was adopted reservation-wide in 2009. Local medical providers have increased awareness and familiarity with SCID. Nearly 40% of Native American patients were treated at two centers through long-standing community-based partnerships that may have allowed for early diagnosis and treatment even prior to NBS implementation. In addition, events such as second transplant and death may have occurred beyond 5 years, which was the benchmark for assessing outcomes. For example, in cases of unconditioned HCT, which successfully rescues patients short-term, poor long-term immune reconstitution frequently occurs (2, 16, 17).

Health equity interventions typically focus on individual and interpersonal factors, which limit sustained improvements (18). In contrast, structural and public health interventions, such as NBS, can have a profound impact by addressing multilevel barriers. Examples of interventions that successfully eliminated health disparities are sparse; one example, the Earned Income Tax Credit, aimed to increase individual wealth among low-income working families and had wide-ranging benefits, including increased prenatal care, reduction in low birth weight among low-income African American newborns, and enhanced child nutrition (19, 20).

NBS has been accompanied by centralized review by experts and strengthened referral pathways, allowing for earlier institution of protective measures for infants with SCID, including isolation, breastfeeding guidance, infection prophylaxis, immunoglobulin administration, and definitive treatment (6, 21). Following NBS, the median age at HCT did not vary by race or ethnicity, which suggests that NBS and subsequent follow-up for abnormal T cell receptor excision circles (TREC) screening are accessible to all patients regardless of race and ethnicity in our cohort. Moreover, previous data suggest universal screening across all 50 states allows for equitable access to TREC screening (8). Younger age at HCT led to improved OS for all patients with SCID, as previously described (9), and OS among Black patients improved notably following the introduction of NBS. The impact of NBS on domains other than time to HCT and infection requires further investigation across racial and ethnic groups.

NBS spans myriad diseases, and thus, the impact of NBS we show here has implications for infant morbidity and mortality beyond patients with SCID. However, NBS alone does not uniformly eliminate disparities across all screened conditions, particularly when physiologic variability in screening biomarkers (22) or differences in downstream access to care (23) contribute to inequities, highlighting that the equity-promoting impact of NBS depends not only on detection, but also on the structure and timeliness of follow-up care. Consideration of expansion of SCID NBS to other countries together with building HCT capacity has the potential to save many lives and to reduce disparities worldwide. Our findings have broad implications—the impact of NBS demonstrates the potential utility of systematic early detection, which has a role in a wide range of different diseases.

This PIDTC dataset is compiled from a large consortium of tertiary-care centers across the United States and Canada, and the near-complete capture of consecutive patients makes the data generalizable across these countries. However, patients with SCID who died prior to diagnosis or HCT or those who were treated outside of these centers were not evaluated in this cohort. Such patients likely have the greatest barriers accessing healthcare; we may have underestimated the impact of access to care as a result. Indeed, SCID incidence estimates prior to SCID NBS were half the actual incidence once screening was in place (6).

The combination of race and ethnicity data with comprehensive clinical data from baseline prior to HCT through many years of follow-up, including clinical history, treatment, genetic data, and laboratory results, yields a rare opportunity to assess the interactions between biological and social determinants of outcome. Race and ethnicity data reflect approaches to data collection at the time—abstraction from health records earlier and self-report more recently. There is thus potential for misclassification of race and ethnicity in some patients. Measures of SES and access to care (such as health insurance type) were not available, raising concern for unmeasured confounding and misattribution of effects to race and ethnicity that might be more accurately explained by SES. Current PIDTC studies are now using self-report of race, ethnicity, and SES from surveys to allow for more accurate assessment. The small numbers of patients within some race and ethnicity categories (specifically Asian/PI patients) coupled with rare predictor variables of interest, such as genotype and donor type, likely reduced statistical power to detect differences.

In summary, this study within a large U.S. and Canadian combined retrospective and prospective cohort emphasizes the important racial and ethnic disparities that exist in survival following nonsibling HCT among patients with SCID. The integration of racial and ethnic data with extensive clinical information provides a robust platform for investigating the interplay between biological and social determinants of survival. The higher rates of favorable genotypes (*IL2RG/JAK3* in Black patients and *IL2RG* in Asian/PI patients), and the associated poorer survival outcomes, present a paradox that warrants further exploration. The enduring impact of donor choice on survival outcomes, even in the contemporary era, also merits future study. The implementation of NBS for SCID stands out as a beacon of progress, contributing to equalizing survival between Black and NH White patients. The remarkable success of NBS not only provides direct benefits to SCID patients but also serves as an exemplar for other health interventions, illustrating the far-reaching impact that systemic changes can have on public health. Ensuring the widespread adoption of NBS remains a critical area for advocacy with the potential to create substantial improvements in health outcomes for diverse populations globally.

## Materials and methods

### Study population

Patients with a diagnosis of typical or leaky SCID or Omenn syndrome who received an allogeneic HCT between 1982 and 2020 and enrolled in PIDTC prospective (NCT01346150/6901) or retrospective (NCT01186913/6902) natural history protocols from 34 U.S. and Canadian centers were included. This study was conducted in accordance with the Declaration of Helsinki and was approved by Institutional Review Boards at each participating center. Written informed consent was obtained from participants’ legal guardians as required by local institutional policies. Patients who received enzyme therapy prior to HCT or gene therapy were excluded due to these therapies’ relationship with underlying mechanisms of disparities including time to transplant and infection risk. HCT was performed according to each treating center’s standard practice. Each center entered clinical and laboratory data into standardized case report forms in a centralized database. The single, initial finding (“trigger”) that led to immunologic testing and confirmation of the SCID diagnosis was classified as follows: (1) FH; (2) NBS; or (3) clinical illness (including infections) (9). The following covariates were evaluated: age at treatment, decade of treatment, infection at the time of HCT, presence of transplacental maternal engraftment, maternal GVHD prior to HCT, and genotype, as well as graft and donor source, conditioning regimen, GVHD prophylaxis, and serotherapy (as previously defined) (2).

### Exposure

The primary exposure of interest was a composite of race and ethnicity collected from the medical record (which may or may not reflect self-reported data depending on the era and institution). NH White patients served as the reference group compared with Hispanic, NH Black, NH Asian/PI, NH Native American, NH Other/Unknown (defined in keeping with National Institutes of Health [NIH] enrollment reporting).

### Outcomes

The primary outcome of interest was 5-year OS. Secondary outcomes included acute and chronic GVHD, need for a subsequent treatment (an additional HSCT from a different donor or from the same donor with conditioning), and 5-year EFS (defined as survival without second HCT). Immune reconstitution and causes of death were also explored by race and ethnicity.

### Statistical analysis

In univariate analysis, patient-, disease-, donor-, and transplant-related variables were compared between different racial and ethnic groups using chi-square statistics for categorical variables and the Wilcoxon test for continuous variables. Analyses were performed separately for those who received HCT from a MSD given their known favorable outcomes (2, 24). The probability of survival was estimated using the Kaplan–Meier method with the log-rank test used for univariate comparisons. The cumulative incidence of subsequent treatment (at 5 years), grade III–IV acute GVHD (at 180 days), and chronic GVHD (at 2 years) were estimated using the cumulative incidence function to allow for competing risks. Cox proportional hazards regression models were used for multivariable analyses, controlling for patient-, disease-, donor-, and transplant-related variables previously shown to be associated with survival, including conditioning, infection status, and decade of HCT. Covariates were retained in the final model using bidirectional stepwise selection based on a P value < 0.05 (and the following confounders were ultimately included in the final multivariable model: age at HCT, conditioning, donor type, SCID genotype, infection status, and decade of HCT). For categorical variables with missing data, a separate missing group was included in the models.

To evaluate NBS as an effect modifier of disparities, analyses stratified by trigger for diagnosis were performed for NH White, Black, and Hispanic populations. Limited numbers precluded these analyses among Asian/PI and Native American populations. To control for the potential impact of changes in HCT over time, a sensitivity analysis was performed in which stratified analyses were restricted to patients diagnosed between 2010 and 2020 (when NBS for SCID was introduced). Active infection pre-HCT (vs. resolved vs. no infection) and time to HCT by race and ethnicity were also stratified by trigger to investigate hypothesized mechanisms.

### Trial registration

This trial was registered at www.clinicaltrials.gov as #NCT01346150 and #NCT01186913.

### Online supplemental material

Supplementary materials include additional tables and figures supporting the main analyses. Figs. S1, S2, and S3 display additional survival analyses, including MSD outcomes and sensitivity analyses restricted to the contemporary era. Fig. S1 shows OS among MSD transplants. Fig. S2 shows EFS among MSD transplants. Fig. S3 shows sensitivity analyses evaluating diagnosis trigger restricted to the contemporary era. Table S1 presents the multivariable model for EFS. Table S2 details posttransplant outcomes, including causes of death and GVHD. Table S3 summarizes transplant characteristics across racial and ethnic groups for the entire cohort including patients who received HCT from a MSD, and Table S4 provides immune reconstitution data at 6 mo after transplant.

## Supplementary Material

Table S1 shows multivariable model for EFS (EFS defined by need for second transplant).

Table S2 shows posttransplant events (causes of death, subsequent treatment, aGVHD, cGVHD) by race and ethnicity among non-MSD transplants.

Table S3 shows transplant characteristics (including MSD).

Table S4 shows (a) posttransplant immune reconstitution at 6 mo after HCT (including MSD) by race and ethnicity and (b) posttransplant immune reconstitution at 6 mo after HCT (restricted to non-MSD) by race and ethnicity.

## Data Availability

The study data will be available upon request from the PIDTC after publication. Individual participant data (de-identified) and the data dictionary will be provided upon approval of a research proposal and executed data sharing agreement. The protocol, analysis plan, and informed consent documents are also available upon request.

## References

[1] Griffith, L.M., M.J. Cowan, L.D. Notarangelo, D.B. Kohn, J.M. Puck, W.T. Shearer, L.M. Burroughs, T.R. Torgerson, H. Decaluwe, E. Haddad, and Workshop Participants. 2016. Primary immune deficiency treatment consortium (PIDTC) update. J. Allergy Clin. Immunol. 138:375–385. 10.1016/j.jaci.2016.01.051 27262745 PMC4986691

[2] Pai, S.-Y., B.R. Logan, L.M. Griffith, R.H. Buckley, R.E. Parrott, C.C. Dvorak, N. Kapoor, I.C. Hanson, A.H. Filipovich, S. Jyonouchi, . 2014. Transplantation outcomes for severe combined immunodeficiency, 2000–2009. N. Engl. J. Med. 371:434–446. 10.1056/NEJMoa1401177 25075835 PMC4183064

[3] Haddad, E., B.R. Logan, L.M. Griffith, R.H. Buckley, R.E. Parrott, S.E. Prockop, T.N. Small, J. Chaisson, C.C. Dvorak, M. Murnane, . 2018. SCID genotype and 6-month posttransplant CD4 count predict survival and immune recovery. Blood. 132:1737–1749. 10.1182/blood-2018-03-840702 30154114 PMC6202916

[4] Lankester, A.C., B. Neven, N. Mahlaoui, E.G.J. von Asmuth, V. Courteille, M. Alligon, M.H. Albert, I.B. Serra, P. Bader, D. Balashov, . 2022. Hematopoietic cell transplantation in severe combined immunodeficiency: The SCETIDE 2006-2014 European cohort. J. Allergy Clin. Immunol. 149:1744–1754.e8. 10.1016/j.jaci.2021.10.017 34718043

[5] Singh, S., J. Ojodu, A.R. Kemper, W.K.K. Lam, and S.D. Grosse. 2023. Implementation of newborn screening for conditions in the United States first recommended during 2010-2018. Int. J. Neonatal. Screen. 9:20. 10.3390/ijns9020020 37092514 PMC10123615

[6] Kwan, A., R.S. Abraham, R. Currier, A. Brower, K. Andruszewski, J.K. Abbott, M. Baker, M. Ballow, L.E. Bartoshesky, F.A. Bonilla, . 2014. Newborn screening for severe combined immunodeficiency in 11 screening programs in the United States. JAMA. 312:729–738. 10.1001/jama.2014.9132 25138334 PMC4492158

[7] Currier, R., and J.M. Puck. 2021. SCID newborn screening: What we’ve learned. J. Allergy Clin. Immunol. 147:417–426. 10.1016/j.jaci.2020.10.020 33551023 PMC7874439

[8] Bedford, S., and K. Vachuska. 2024. Assessing interstate racial and socioeconomic disparities in newborn screening policies in the United States. Front. Public Health. 12:1310516. 10.3389/fpubh.2024.1310516 38741907 PMC11089229

[9] Thakar, M.S., B.R. Logan, J.M. Puck, E.A. Dunn, R.H. Buckley, M.J. Cowan, R.J. O'Reilly, N. Kapoor, L.F. Satter, S.-Y. Pai, . 2023. Measuring the effect of newborn screening on survival after haematopoietic cell transplantation for severe combined immunodeficiency: A 36-year longitudinal study from the primary immune deficiency treatment consortium. Lancet. 402:129–140. 10.1016/S0140-6736(23)00731-6 37352885 PMC10386791

[10] Majhail, N.S., S. Nayyar, M.E.B. Santibañez, E.A. Murphy, and E.M. Denzen. 2012. Racial disparities in hematopoietic cell transplantation in the United States. Bone Marrow Transpl. 47:1385–1390. 10.1038/bmt.2011.214 PMC3848311 22056642

[11] Hong, S., and N.S. Majhail. 2021. Increasing access to allotransplants in the United States: The impact of race, geography, and socioeconomics. Hematol. Am Soc. Hematol. Educ. Program. 2021:275–280. 10.1182/hematology.2021000259 PMC8791157 34889386

[12] Brown Speights, J.S., S.S. Goldfarb, B.A. Wells, L. Beitsch, R.S. Levine, and G. Rust. 2017. State-level progress in reducing the Black–White infant mortality gap, United States, 1999–2013. Am. J. Public Health. 107:775–782. 10.2105/AJPH.2017.303689 28323476 PMC5388953

[13] Khera, N., S. Ailawadhi, R. Brazauskas, J. Patel, B. Jacobs, C. Ustun, K. Ballen, M.B. Abid, M.A. Diaz Perez, A.S. Al-Homsi, . 2024. Trends in volumes and survival after hematopoietic cell transplantation in racial/ethnic minorities. Blood Adv. 8:3497–3506. 10.1182/bloodadvances.2023012469 38661372 PMC11260842

[14] Gragert, L., M. Eapen, E. Williams, J. Freeman, S. Spellman, R. Baitty, R. Hartzman, J.D. Rizzo, M. Horowitz, D. Confer, and M. Maiers. 2014. HLA match likelihoods for hematopoietic stem-cell grafts in the U.S. registry. N. Engl. J. Med. 371:339–348. 10.1056/nejmsa1311707 25054717 PMC5965695

[15] Kwan, A., D. Hu, M. Song, H. Gomes, D.R. Brown, T. Bourque, D. Gonzalez-Espinosa, Z. Lin, M.J. Cowan, and J.M. Puck. 2015. Successful newborn screening for SCID in the Navajo Nation. Clin. Immunol. 158:29–34. 10.1016/j.clim.2015.02.015 25762520 PMC4420660

[16] Cavazzana-Calvo, M., F. Carlier, F. Le Deist, E. Morillon, P. Taupin, D. Gautier, I. Radford-Weiss, S. Caillat-Zucman, B. Neven, S. Blanche, . 2007. Long-term T-cell reconstitution after hematopoietic stem-cell transplantation in primary T-cell–immunodeficient patients is associated with myeloid chimerism and possibly the primary disease phenotype. Blood. 109:4575–4581. 10.1182/blood-2006-07-029090 17272510

[17] Schuetz, C., B. Neven, C.C. Dvorak, S. Leroy, M.J. Ege, U. Pannicke, K. Schwarz, A.S. Schulz, M. Hoenig, M. Sparber-Sauer, . 2014. SCID patients with ARTEMIS vs RAG deficiencies following HCT: Increased risk of late toxicity in ARTEMIS-deficient SCID. Blood. 123:281–289. 10.1182/blood-2013-01-476432 24144642 PMC3953035

[18] Brown, A.F., G.X. Ma, J. Miranda, E. Eng, D. Castille, T. Brockie, P. Jones, C.O. Airhihenbuwa, T. Farhat, L. Zhu, and C. Trinh-Shevrin. 2019. Structural interventions to reduce and eliminate health disparities. Am. J. Public Health. 109:S72–S78. 10.2105/AJPH.2018.304844 30699019 PMC6356131

[19] Hamad, R., and D.H. Rehkopf. 2015. Poverty, pregnancy, and birth outcomes: A study of the earned income Tax Credit. Paediatr. Perinat. Epidemiol. 29:444–452. 10.1111/ppe.12211 26212041 PMC4536129

[20] Hoynes, H., D. Miller, and D. Simon. 2015. Income, the earned income tax credit, and infant health. Am. Econ. J. Econ. Policy. 7:172–211. 10.1257/pol.20120179

[21] Dorsey, M.J., N.A.M. Wright, N.S. Chaimowitz, B.J. Dávila Saldaña, H. Miller, M.D. Keller, M.S. Thakar, A.J. Shah, R. Abu-Arja, J. Andolina, . 2021. Infections in infants with SCID: Isolation, infection screening, and prophylaxis in PIDTC centers. J. Clin. Immunol. 41:38–50. 10.1007/s10875-020-00865-9 33006109 PMC8388237

[22] Peng, G., Y. Tang, N. Gandotra, G.M. Enns, T.M. Cowan, H. Zhao, and C. Scharfe. 2020. Ethnic variability in newborn metabolic screening markers associated with false-positive outcomes. J. Inherit. Metab. Dis. 43:934–943. 10.1002/jimd.12236 32216101 PMC7540352

[23] McColley, S.A., S.L. Martiniano, C.L. Ren, M.K. Sontag, K. Rychlik, L. Balmert, A. Elbert, R. Wu, and P.M. Farrell. 2023. Disparities in first evaluation of infants with cystic fibrosis since implementation of newborn screening. J. Cyst. Fibros. 22:89–97. 10.1016/j.jcf.2022.07.010 35871976

[24] Heimall, J., B.R. Logan, M.J. Cowan, L.D. Notarangelo, L.M. Griffith, J.M. Puck, D.B. Kohn, M.A. Pulsipher, S. Parikh, C. Martinez, . 2017. Immune reconstitution and survival of 100 SCID patients post-hematopoietic cell transplant: A PIDTC natural history study. Blood. 130:2718–2727. 10.1182/blood-2017-05-781849 29021228 PMC5746165

