# Supplementary material for: Newborn screening reduces survival disparities in SCID after stem cell transplant: A PIDTC report
Source: J Hum Immun. 2026 Jul 7;2(5):e20250231. doi: 10.70962/jhi.20250231 (PMC13340543; doi:10.70962/jhi.20250231)
Supplement: Table S1 — shows multivariable model for EFS (EFS defined by need for second transplant). [file jhi_20250231_tables1.docx]

***Supplemental Table 1. Multivariable model for EFS (EFS defined by need for second transplant)***

| Race and Ethnicity | **Median EFS** | **aHR (95% CI)** | **p value** | **Overall P value** |
| --- | --- | --- | --- | --- |
| Non-Hispanic White | 69.9 (62.1-71.4) | ref |  | 0.006 |
| Hispanic | 62.4 (54.6-69.3) | 1.83 (1.27, 2.63) | **0.001** |  |
| Asian/Pacific Islander | 52.4 (40.5-63.0) | 1.24 (0.92, 1.68) | 0.15 |  |
| Black | 62.1 (44.6-75.6) | 1.41 (0.80, 2.48) | 0.24 |  |
| Native American | 60.4 (40.5-75.5) | 0.78 (0.44, 1.40) | 0.41 |  |
| Other/Unknown | 59.7 (45.0-71.7) | 1.63 (1.04, 2.55) | **0.03** |  |

*Final model accounting for age at treatment, infection status, conditioning, donor source, genotype, and decade of treatment
