# Supplementary material for: Newborn screening reduces survival disparities in SCID after stem cell transplant: A PIDTC report
Source: J Hum Immun. 2026 Jul 7;2(5):e20250231. doi: 10.70962/jhi.20250231 (PMC13340543; doi:10.70962/jhi.20250231)
Supplement: Table S2 — shows posttransplant events (causes of death, subsequent treatment, aGVHD, cGVHD) by race and ethnicity among non-MSD transplants. [file jhi_20250231_tables2.docx]

***Supplemental Table 2. Post Transplant Events (causes of death, subsequent treatment, aGVHD, cGVHD) by Race and Ethnicity among non-MSD transplants***

| **Outcome** | **NH White**  **N=421 (%)** | **Hispanic**  **N=174 (%)** | **Black**  **N=77 (%)** | **Asian/PI**  **N=37 (%)** | **Nat. American N=33 (%)** | **Unknown**  **N=54 (%)** | **P Value** |
| --- | --- | --- | --- | --- | --- | --- | --- |
| Causes of Death | | | | | | | |
| Infection | 52 (52.5) | 29 (59.2) | 21 (63.6) | 5 (35.7) | 4 (44.4) | 11 (55.0) | 0.54 |
| Respiratory Failure | 15 (15.2) | 7 (14.2) | 4 (12.1) | 3 (14.3) | 1 (11.1) | 0 (0.0) |  |
| Other Organ Failure | 8 (8.1) | 6 (12.2) | 1 (3.0) | 4 (28.6) | 1 (11.1) | 3 (15.0) |  |
| Graft-versus-Host Disease | 5 (5.1) | 1 (2.0) | 2 (6.1) | 1 (7.1) | 0 (0.0) | 2 (10.0) |  |
| Other/Unknown | 19 (19.2) | 6 (12.2) | 5 (15.1) | 1 (7.1) | 3 (33.3) | 4 (20.0) |  |
| Cumulative incidence of subsequent Tx at 5 years (CI) | 17.3  (13.8-21.2) | 18.1  (12.7-24.2) | 15.8  (8.6-25.0) | 10.8  (3.4-23.3) | 20.8  (8.1-37.6) | 15.8  (7.2-27.4) | 0.77 |
| Cumulative incidence of Acute GVHD grade II - IV at Day 180 (CI) | 22.3  (18.4-26.5) | 24.4  (18.2-31.1) | 23.0  (14.1-33.1) | 31.4  (16.9-47.1) | 27.6  (12.8-44.6) | 14.3  (6.2-25.7) | 0.50 |
| Cumulative incidence of Acute GVHD grade III - IV at Day 180 (CI) | 9.8  (7.2-13.0) | 14.3  (9.5-20.0) | 8.1  (3.3-15.8) | 11.4  (3.5-24.5) | 10.3  (2.6-24.6) | 4.1  (0.7-12.4) | 0.36 |
| Cumulative incidence of Chronic GVHD at 2 years (CI) | 16.8  (13.2-20.8) | 17.6  (12.3-23.6) | 13.9  (7.1-23.0) | 11.1  (3.4-23.9) | 3.5  (0.2-15.3) | 10.3  (3.1-22.5) | 0.30 |

GVHD: Graft-versus-Host Disease
