# Supplementary material for: Newborn screening reduces survival disparities in SCID after stem cell transplant: A PIDTC report
Source: J Hum Immun. 2026 Jul 7;2(5):e20250231. doi: 10.70962/jhi.20250231 (PMC13340543; doi:10.70962/jhi.20250231)
Supplement: Table S3 — shows transplant characteristics (including MSD). [file jhi_20250231_tables3.docx]

**Supplemental Table 3. Transplant Characteristics (including MSD)**

| **Characteristic** | Total N=925 | NH White N=477 | Hispanic N=201 | Black N=96 | Asian/PI N=43 | Nat.American N=42 | Unknown/Other N=66 | P value |
| --- | --- | --- | --- | --- | --- | --- | --- | --- |
| **Graft Source** | | | | | | | | <0.001 |
| Bone marrow | 636 (68.8) | 361 (75.7) | 108 (53.7) | 70 (72.9) | 20 (46.5) | 27 (64.3) | 50 (75.8) |  |
| Cord blood | 136 (14.7) | 64 (13.4) | 41 (20.4) | 13 (13.5) | 11 (25.6) | 1 (2.4) | 6 (9.1) |  |
| Peripheral blood stem cells | 152 (16.4) | 52 (10.9) | 52 (25.9) | 13 (13.5) | 11 (25.6) | 14 (33.3) | 10 (15.2) |  |
| Cord + Marrow | 1 (0.1) | 0 (0.0) | 0 (0.0) | 0 (0.0) | 1 (2.3) | 0 (0.0) | 0 (0.0) |  |
| **Conditioning Regimen** | | | | | | | | <0.001 |
| None | 402 (43.5) | 236 (49.5) | 60 (29.9) | 57 (59.4) | 13 (30.2) | 12 (28.6) | 24 (36.4) |  |
| Immunosuppression | 134 (14.5) | 59 (12.4) | 33 (16.4) | 9 (9.4) | 3 (7.0) | 20 (47.6) | 10 (15.2) |  |
| Reduced intensity | 164 (17.7) | 77 (16.1) | 40 (19.9) | 17 (17.7) | 12 (27.9) | 2 (4.8) | 16 (24.2) |  |
| Myeloablative | 221 (23.9) | 103 (21.6) | 67 (33.3) | 13 (13.5) | 14 (32.6) | 8 (19.0) | 16 (24.2) |  |
| Unknown | 4 (0.4) | 2 (0.4) | 1 (0.5) | 0 (0.0) | 1 (2.3) | 0 (0.0) | 0 (0.0) |  |
| **Donor Source** | | | | | | | | 0.28 |
| HLA-identical sibling | 129 (13.9) | 56 (11.7) | 27 (13.4) | 19 (19.8) | 6 (14.0) | 9 (21.4) | 12 (18.2) |  |
| HLA-matched other relative | 37 (4.0) | 21 (4.4) | 8 (4.0) | 3 (3.1) | 1 (2.3) | 1 (2.4) | 3 (4.5) |  |
| HLA-matched unrelated | 117 (12.6) | 71 (14.9) | 25 (12.4) | 5 (5.2) | 5 (11.6) | 3 (7.1) | 8 (12.1) |  |
| HLA-mismatched relative | 481 (52.0) | 254 (53.2) | 99 (49.3) | 53 (55.2) | 18 (41.9) | 26 (61.9) | 31 (47.0) |  |
| HLA-mismatched unrelated | 146 (15.8) | 66 (13.8) | 38 (18.9) | 16 (16.7) | 12 (27.9) | 3 (7.1) | 11 (16.7) |  |
| **GVHD prophylaxis** | | | | | | | | 0.47 |
| Ex vivo T cell depletion | 505 (54.6) | 272 (57.0) | 104 (51.7) | 57 (59.4) | 17 (39.5) | 25 (59.5) | 30 (45.5) |  |
| IS + ATG/ Alemtuzumab | 247 (26.7) | 121 (25.4) | 57 (28.4) | 19 (19.8) | 15 (34.9) | 12 (28.6) | 23 (34.8) |  |
| IS only | 119 (12.9) | 57 (11.9) | 31 (15.4) | 12 (12.5) | 7 (16.3) | 3 (7.1) | 9 (13.6) |  |
| None | 54 (5.8) | 27 (5.7) | 9 (4.5) | 8 (8.3) | 4 (9.3) | 2 (4.8) | 4 (6.1) |  |
| **Serotherapy** | | | | | | | | <0.001 |
| None | 520 (56.2) | 292 (61.2) | 96 (47.8) | 68 (70.8) | 18 (41.9) | 16 (38.1) | 30 (45.5) |  |
| ATG | 312 (33.7) | 142 (29.8) | 78 (38.8) | 22 (22.9) | 20 (46.5) | 22 (52.4) | 28 (42.4) |  |
| ATG + Alemtuzumab | 4 (0.4) | 3 (0.6) | 0 (0.0) | 1 (1.0) | 0 (0.0) | 0 (0.0) | 0 (0.0) |  |
| Alemtuzumab | 89 (9.6) | 40 (8.4) | 27 (13.4) | 5 (5.2) | 5 (11.6) | 4 (9.5) | 8 (12.1) |  |

HLA: human leukocyte antigen, IS: immunosuppression, ATG: anti-thymocyte globulin
