# Supplementary material for: Newborn screening reduces survival disparities in SCID after stem cell transplant: A PIDTC report
Source: J Hum Immun. 2026 Jul 7;2(5):e20250231. doi: 10.70962/jhi.20250231 (PMC13340543; doi:10.70962/jhi.20250231)
Supplement: Table S4 — shows (a) posttransplant immune reconstitution at 6 mo after HCT (including MSD) by race and ethnicity and (b) posttransplant immune reconstitution at 6 mo after HCT (restricted to non-MSD) by race and ethnicity. [file jhi_20250231_tables4.docx]

**Supplemental Table 4a. Post-transplant Immune Reconstitution at 6 months post-HCT (including MSD) by Race and Ethnicity**

| **Immune Reconstitution Subset** | **N*** | **Overall** | **NH White** | **Hispanic** | **Black** | **Asian/PI** | **Native American** | **Unknown** | **P value** |
| --- | --- | --- | --- | --- | --- | --- | --- | --- | --- |
| **Median NK cell count (CD16 or CD56)** | 515 | 188 | 181 | 241 | 149 | 398 | 182 | 219 | **0.05** |
| **Median T cell count (CD3)** | 582 | 747 | 725 | 917 | 811 | 702 | 405 | 674 | 0.463 |
| Median Cytotoxic T cell count (CD8) | 562 | 246 | 249 | 257 | 296 | 316 | 141 | 228 | 0.77 |
| Median Helper T cell count (CD4) | 570 | 424 | 404 | 533 | 546 | 469 | 160 | 340 | 0.07 |
| Median Memory T cell count   (CD4 CD45RO) | 231 | 175 | 153 | 280 | 179 | 308 | 60 | 196 | **0.01** |
| Median Naïve T cell count   (CD4 CD45RA) | 249 | 210 | 222 | 374 | 284 | 67 | 0 | 136 | **0.006** |
| Naïve T cell (%) | 248 | 44 | 47 | 54 | 41 | 14 | 0 | 31 | **0.01** |
| **Median B cell count (CD19 or CD20)** | 513 | 403 | 410 | 359 | 724 | 649 | 2 | 369 | **<0.001** |
| Off IVIG (%) | 668 | 15 | 15 | 16 | 22 | 23 | 0 | 13 | 0.11 |

**** Missing data varied by test type among the 925 total patients***

**Supplemental Table 4b. Post-transplant Immune Reconstitution at 6 months post-HCT (restricted to non-MSD) by Race and Ethnicity**

| **Immune Reconstitution Subset** | **N*** | **Overall** | **NH White** | **Hispanic** | **Black** | **Asian/PI** | **Native American** | **Unknown** | **P value** |
| --- | --- | --- | --- | --- | --- | --- | --- | --- | --- |
| **Median NK cell count (CD16 CD56)** | 442 | 182 | 172 | 225 | 170 | 398 | 182 | 228 | 0.06 |
| **Median T cell count (CD3)** | 496 | 706 | 698 | 883 | 708 | 702 | 347 | 640 | 0.45 |
| Median Cytotoxic T cell count (CD8) | 483 | 222 | 232 | 234 | 190 | 189 | 119 | 224 | 0.76 |
| Median Helper T cell count (CD4) | 487 | 410 | 395 | 528 | 427 | 506 | 138 | 375 | 0.16 |
| Median Memory T cell count   (CD4 CD45RO) | 204 | 168 | 152 | 247 | 178 | 270 | 60 | 147 | **0.05** |
| Median Naïve T cell count   (CD4 CD45RA) | 220 | 199 | 216 | 300 | 178 | 62 | 0 | 136 | **0.02** |
| Naïve T cell (%) | 219 | 42 | 46 | 52 | 39 | 10 | 0 | 31 | **0.02** |
| **Median B cell count (CD19 or CD20)** | 440 | 383 | 410 | 344 | 598 | 711 | 2 | 290 | **<0.001** |
| Off IVIG (%) | 464 | 13 | 13 | 13 | 14 | 27 | 0 | 6 | 0.08 |

**** Missing data varied by test type among 796 patients receiving non-MSD transplants***
